# MnasNet-SimAM: An Improved Deep Learning Model for the Identification of Common Wheat Diseases in Complex Real-Field Environments

**DOI:** 10.3390/plants13162334

**Published:** 2024-08-22

**Authors:** Xiaojie Wen, Muzaipaer Maimaiti, Qi Liu, Fusheng Yu, Haifeng Gao, Guangkuo Li, Jing Chen

**Affiliations:** 1 Key Laboratory of the Pest Monitoring and Safety Control of Crops and Forests of the Xinjiang Uygur Autonomous Region, College of Agronomy, Xinjiang Agricultural University, Urumqi 830052, China; adgerx@163.com (X.W.); muzappar0829@163.com (M.M.); 19860936350@163.com (F.Y.); 2 Key Laboratory of Prevention and Control of Invasive Alien Species in Agriculture & Forestry of the North-Western Desert Oasis, Ministry of Agriculture and Rural Affairs, Urumqi 830052, China; 3 Institute of Plant Protection, Xinjiang Academy of Agricultural Science, Urumqi 830091, China; xghf20044666@163.com (H.G.); lgk0808@163.com (G.L.); 4 Key Laboratory of Integrated Pest Management on Crop in Northwestern Oasis, Ministry of Agriculture and Rural Affairs, Urumqi 830091, China

**Keywords:** deep learning, remote sensing, wheat disease, MnasNet, attention mechanism

## Abstract

Deep learning approaches have been widely applied for agricultural disease detection. However, considerable challenges still exist, such as low recognition accuracy in complex backgrounds and high misjudgment rates for similar diseases. This study aimed to address these challenges through the detection of six prevalent wheat diseases and healthy wheat in images captured in a complex natural context, evaluating the recognition performance of five lightweight convolutional networks. A novel model, named MnasNet-SimAM, was developed by combining transfer learning and an attention mechanism. The results reveal that the five lightweight convolutional neural networks can recognize the six different wheat diseases with an accuracy of more than 90%. The MnasNet-SimAM model attained an accuracy of 95.14%, which is 1.7% better than that of the original model, while only increasing the model’s parameter size by 0.01 MB. Additionally, the MnasNet-SimAM model reached an accuracy of 91.20% on the public Wheat Fungi Diseases data set, proving its excellent generalization capacity. These findings reveal that the proposed model can satisfy the requirements for rapid and accurate wheat disease detection.

## 1. Introduction

Wheat is the world’s most important cereal crop, being directly intertwined with humanity’s survival and advancement [1]. Common wheat diseases include wheat rust [2,3,4], wheat powdery mildew [5], wheat smut [6], and wheat scab [7]. These diseases significantly reduce the quality and yield of wheat, resulting in substantial economic losses. Therefore, the rapid and accurate detection and identification of wheat diseases are vital measures to ensure healthy wheat growth and safeguard agricultural security [8].

Deep learning (DL) approaches have been frequently employed in identification tasks in the agricultural field and have produced remarkable outcomes, allowing for high identification accuracy at a relatively low cost [9]. However, one of the biggest challenges when using DL for agricultural recognition tasks is image recognition accuracy. Real-world agricultural production images are often affected by complex backgrounds, adverse weather conditions, focus blurring, occlusion, and the presence of irrelevant objects [10]. Additionally, the image quality can be significantly impacted by the image’s size, position, and shape, as well as the lighting and shooting conditions. These variables are the primary causes of classification errors. Therefore, improving image recognition capability is a significant issue in environments with complex backgrounds.

At present, attention mechanisms are widely used to improve model performance [11]. Attention mechanisms are a technique used in deep learning to simulate selective attention and weighted processing of input data, similarly to processes in the human visual system [12]. In a traditional neural network model, each input is usually treated equally without difference. However, in the actual task, the input data require different levels of attention for different components or regions. This notion is particularly crucial in agricultural classification tasks with complex backgrounds. When identifying specific crop diseases, it is crucial to focus on regions that exhibit distinct disease symptoms. Therefore, an attention mechanism is needed to efficiently allocate attention to relevant areas through autonomous learning and weight adjustment. In addition, through the use of Grad-CAM, attention mechanisms are also easy to interpret, effectively describing the key features or areas emphasized by the model to facilitate the decision-making process [13]. For large agricultural objects, deep learning models are now superior to other classification methods; however, their classification performance may fall short of expectations in the initial stages of symptom appearance and when considering diseases with similar symptoms. Therefore, this disadvantage could be somewhat compensated for with the introduction of an attention mechanism.

Attention mechanisms have been introduced into the field of computer vision, imitating the ability of the human visual system to focus on salient regions in complex scenes and categorize those regions according to various approaches, such as channel attention, spatial attention, temporal attention, branch attention, and so on [14]. Spatial attention and channel attention are often used in deep learning. Their main difference lies in whether the mechanism focuses on a specific spatial region of the input data or a specific channel when dealing with images or feature maps. The channel attention mechanism enhances the model’s ability to use multi-channel feature information and representations through allowing for weighted processing and selective attention to input channel dimensions. Meanwhile, the spatial attention mechanism helps to carry out weighted processing and apply selective attention to the input spatial dimensions, and can effectively use the feature information of different locations to improve the perceptual range and accuracy of the model. Common attention mechanisms include the squeeze-and-excitation (SE) module [15], channel attention (CA) module [16], efficient channel attention (ECA) module [17], convolutional block attention module (CBAM) [18], and simple parameter-free attention module (SimAM) [19]. Plug-and-play attention mechanisms can be easily integrated into pre-existing models, allowing for significant improvements in accuracy with very few additional parameters. At present, integrating spatial attention, channel attention, or both, is a major method for improving model performance [20].

In order to identify crop diseases swiftly and accurately, Genaev et al. [21] proposed a method for the recognition of five fungal diseases of wheat shoots based on EfficientNetB0. An approach based on an image hashing algorithm was used to reduce the degradation of the training data. The highest accuracy of the model regarding the used data set was 94.20%. Nigam et al. [22] created a data set called WheatRust21 and used a fine-tuned EfficientNetB4 to achieve 99.35% test accuracy on this data set. Nigam et al. [23] combined the attention mechanism with the EfficientNetB0 model to detect the WheatRust21 image data set and obtained a test set accuracy of 98.70%. Cheng et al. [24] proposed a lightweight crop disease image recognition model, DSGIResNet_AFF, based on attention feature fusion. This model was superior to other network models, and its parameters and number of floating point operations were fewer than those of the original model, with an accuracy of 98.30%, which was suitable for mobile devices. Zhao et al. [25] proposed a model called DTL-SE-ResNet50, which integrates the SE module into ResNet50 based on dual-transfer learning to achieve vegetable disease recognition under simple and complex backgrounds, and performed better than the traditional model. The system could identify vegetable diseases quickly with a short detection time and high accuracy, compared with dtl-cam-resnet50 and DTL-SA-ResNet50. A network that deeply integrated the SE module into the ShuffleNetV2 network was constructed by Xu et al. [26]. The accuracy of the model was 4.85% higher than that of the original model. Yang et al. [27] established a model named DGLNet to solve the problems related to background noise and the dispersed distribution of disease symptoms in real environments. The model combined the Global Attention Module (GAM) and the Dynamic Representation Module (DRM). The results showed that the recognition accuracy of DGLNet reached 99.82% and 99.71% on the two plant disease data sets, respectively, outperforming state-of-the-art methods. Chen et al. [28] proposed a novel domain adaptive image recognition method called simple domain adaptation network (SDAN), which combines channel and location attention modules for disease recognition in rice with a small number of samples.

The above studies significantly proved that the use of an attention mechanism could improve the accuracy of plant disease recognition models. In this study, a lightweight convolutional neural network for wheat disease based on near-ground remote sensing data, named MnasNet-SimAM, is proposed to solve the persistent problem of difficulty in recognizing crop disease in real complex environments. The SimAM module is used to extract depth features, focus on the disease locations, and avoid redundant information. In addition, the training speed and recognition ability of the network are improved through the use of improved activation functions and normalization. The main contributions of this research are outlined below:

1. The effectiveness of five lightweight convolutional neural networks to identify six common wheat diseases and healthy wheat is explored, based on two optimizers and three learning rate scheduling strategies.
2. The influence of different values of λ in the SimAM module on model recognition accuracy is studied, and the performance of the improved model is verified through visualization of the model results. Grad-Cam is used to compare the effects of different attention mechanisms in MnasNet.
3. The influence of agricultural pre-training of weights on the model’s dual transfer learning is analyzed.
4. The generalization ability of MnasNet-SimAM on public data sets is validated.

## 2. Materials and Methods

### 2.1. Image Acquisition

**Figure 1:**
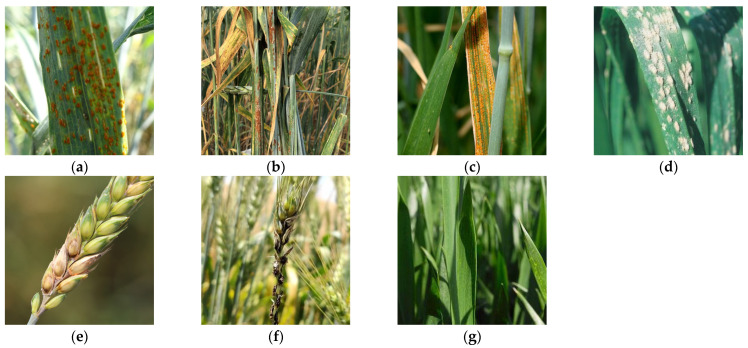
Images showing typical wheat disease symptoms in natural environments: (**a**) wheat leaf rust; (**b**) wheat stem rust; (**c**) wheat stripe rust; (**d**) wheat powdery mildew; (**e**) FHB in wheat; (**f**) wheat smut; and (**g**) healthy wheat.

Wheat disease images, including wheat stripe rust, leaf rust, stem rust, smut, mildew, Fusarium head blight (FHB), and healthy wheat leaves, were obtained from three different sources: (1) field photography, (2) public data sets, and (3) web crawling. For the field photography, during 2022–2023, we collected different images in two main wheat-producing areas of Xinjiang, Yili Kazak Autonomous Prefecture and Bayingolin Mongol Autonomous Prefecture. The collection time was from 10 a.m. to 6 p.m. on a sunny day. The images were taken in automatic exposure mode using a 48 megapixel mobile phone camera with the natural background of the field. All obtained images were in JPG format. Some wheat disease images were obtained from a public website (https://aistudio.baidu.com/). Figure 1 shows examples of wheat disease images with complex backgrounds. A total of 4677 images were collected, including 2117 images captured on-site, 895 images obtained from web sources, and 1665 images acquired from public data sets.

### 2.2. Image Preprocessing

**Figure 2:**
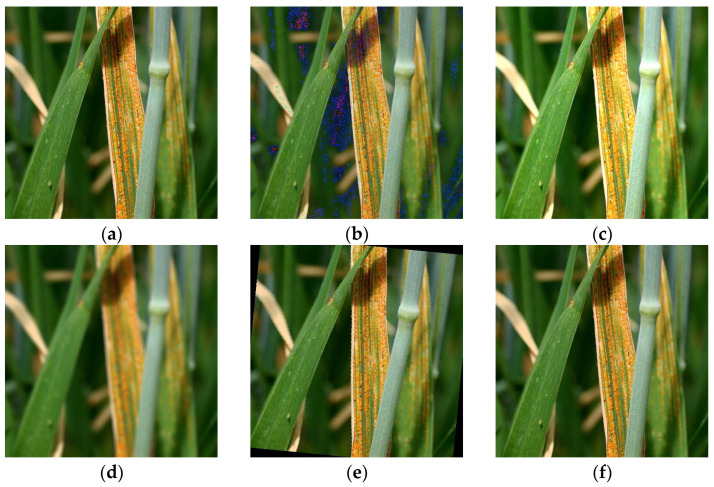
Data enhancement method: (**a**) original image; (**b**) increased Gaussian noise; (**c**) random brightness adjustment; (**d**) Gaussian blur; (**e**) random rotation; and (**f**) random scaling.

Images with poor pixels were removed, and 500 images of each type of disease were selected from the original images. In this way, a total of 3500 images were obtained. In order to prevent overfitting of the model, random data enhancement was carried out on the images, including random rotation, random scaling, brightness adjustment, Gaussian blur, and Gaussian noise addition (Figure 2). After enhancement, a total of 7000 images were obtained, and the data set was divided into training, verification, and test sets in a ratio of 7:2:1 [29]. The sample distribution of the data sets is presented in Table 1. The images were normalized and scaled to 224 × 224 pixels, in order to reduce model overfitting and accelerate model convergence before training.

### 2.3. Attention Mechanism

**Figure 3:**
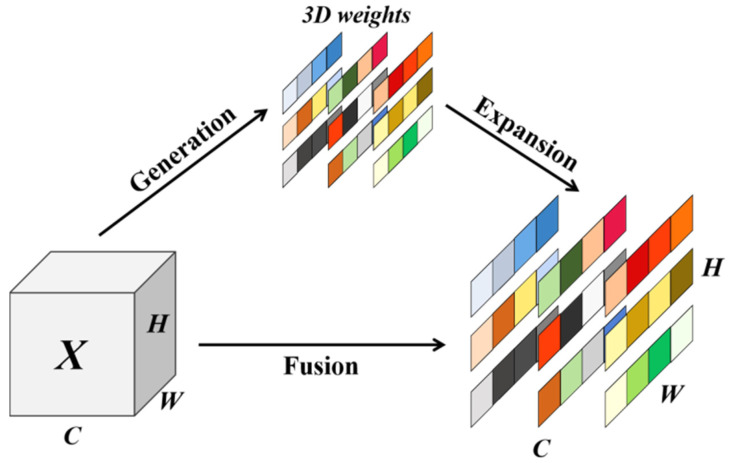
SimAM: Full 3D weights for attention.

The attention mechanism concept is inspired by the ability of the human brain to focus more attention on the important information in input data. As such, the use of an attention mechanism enables a model to efficiently process large amounts of data and extract critical information to improve its performance and generalization ability, better capturing complex relationships in the data [30]. Attention mechanisms can help models to resolve information imbalances in input data, highlight key parts, and reduce attention to noisy or irrelevant information, especially in complex contexts such as disease identification [31,32]. In complex classification tasks, the input data may contain a large amount of redundant or secondary information. The use of an attention mechanism allows the model to better discriminate the critical information from secondary information, improving its perception of crucial information, enhancing its robustness to input data and making it more sensitive to small changes in the input data. A 3D attention module has been proposed, and an energy function was designed to calculate the attention weights in 2021, named the simple-parameter-free attention module [19]. As a 3D attention mechanism, SimAM considers the correlation of spatial and channel dimensions simultaneously through feature mapping of the feature layer without adding parameters to the original network, as depicted in Figure 3. The individual neurons estimate the importance, through which SimAM can calculate the attention weights. In neuroscience, information-rich neurons usually exhibit firing patterns different from those of their surrounding neurons. Moreover, activating neurons usually inhibit peripheral neurons, in a process known as spatial inhibition. In other words, neurons with spatial inhibition should receive higher attention, and the simplest method of finding these neurons is to measure the linear repairability between the target neuron and other neurons. Based on these neuroscientific findings, the following energy function for each neuron was defined in this study (Formula (1) [19]):

(1) $$e_{t}\left(\omega_{t},b_{t},y{,x}_{t}\right)={\left(y_{t}-\hat{t}\right)}^{2}+\frac{1}{M-1}\sum_{i=1}^{M-1}{\left(y_{o}-\hat{x_{i}}\right)}^{2}.$$

Minimizing the above formula is equivalent to linear separability between the training neurons *t* and other neurons in the same channel. For simplicity, binary labeling was used to add the regular terms, and the final energy function is defined as follows (Formula (2) [19]):

(2) $$e_{t}\left(\omega_{t},b_{t},y{,x}_{t}\right)=\frac{1}{M-1}\sum_{i=1}^{M-1}{\left(-1-\left(\omega_{t}x_{i}+b_{t}\right)\right)}^{2}+{\left(1-{(\omega }_{t}t+b_{t})\right)}^{2}+\lambda \omega_{t}^{2},$$

where $\hat{t}$ = $\omega_{t}$t + $b_{t}$ and $\hat{x_{i}}$ = $\omega_{t}x_{i}$ + $b_{t}$ are linear transforms of t and $x_{i}$, respectively, where t and $x_{i}$ are target neurons in a single channel of the input feature $X\in R^{C\times H\times W}$; i is an index over the spatial dimension; M=H×W is the number of neurons on the channel; and $\omega_{t}$ and $b_{t}$ are the weight and bias parameters.

The early Squeeze-and-Excitation (SE) module can adaptively learn the importance of each channel through the introduction of squeeze-and-excitation operations, adjusting the channel contribution in the feature map through dynamic weighting [33]. The SE module uses a global pooling operation to learn the weight vector of the channel dimension, then multiplies the weight vector with the original features to obtain an enhanced feature representation. The Channel Attention (CA) module also introduces a channel dimension attention mechanism, but unlike the SE module, the CA module uses two parallel convolutions to generate attention and multiplies the attention with the original feature to enhance the feature representation. The CA module can capture the relationship between channels more accurately [34]. The Efficient Channel Attention (ECA) module can calculate the attention weight for each position through applying a learnable convolution kernel to the channel dimension [35]. Using the ECA module can reduce the number of parameters and computational complexity of a model, while introducing an attention mechanism to enhance feature representation. The Convolutional Block Attention Module (CBAM) is a module that combines channel attention and spatial attention [36]. First, channel attention is introduced through the SE module, then an attention graph is generated in the spatial dimension using lightweight convolution operations, and finally, the final feature representation is obtained through multiplying the channel attention graph with the space attention graph. The SimAM model is relatively smaller compared to other attention mechanisms, as it does not introduce additional learnable parameters. This is advantageous for inference in the case of limited model size, and the computational process may be more concise and efficient. In addition, the relatively smaller number of model parameters makes it easier to adapt to new task data and reduces the risk of overfitting. The attention calculation of the SimAM module is more transparent and interpretable, due to its parameterless design. However, it also has certain limitations. For example, the expressive ability of the model is limited, as it cannot adapt different tasks through learnable parameters. Adding more parameters to SimAM may improve the model performance for some complex tasks. In this study, in order to explore the performance of SimAM, a comparative experiment was conducted using the above attention mechanism.

### 2.4. Improved MnasNet Architecture

**Figure 4:**
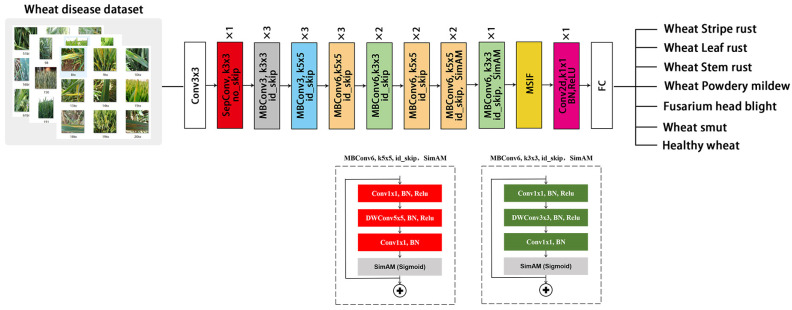
Structure of MnasNet-SimAM.

Dual transfer learning transfers knowledge from a source task to a target task through one or more intermediate tasks and further applies the learned knowledge to the new task, resulting in a better match between the source and target domains [37]. The apple leaf disease data set includes apple scab, apple rust, mixed disease, and healthy leaf images. As there are some similarities between the characteristics of apple leaf and wheat diseases, through pre-training on the apple disease data set, we explored whether the model could enhance its ability to capture wheat disease symptoms. The MnasNet model was improved through adding attention mechanism modules and using the dual transfer learning method. The attention mechanism was integrated into the last three inverted residual networks of MnasNet without changing the backbone network structure. The modified MnasNet-SimAM structure is shown in Figure 4. The steps of combining agricultural dual transfer learning with the attention mechanism were as follows: First, the SimAM module was added to the MnasNet model, which was pre-trained on ImageNet. Then, dual transfer learning was performed on the apple disease data set to obtain new weights for MnasNet-SimAM. Finally, MnasNet-SimAM was trained on the wheat disease data set, and the model was evaluated using the test set. In addition, MnasNet was optimized using other attention mechanisms, and the obtained results were compared.

### 2.5. Evaluation Indicators

The performance evaluation of image classification models often relies on five common indicators: accuracy, precision, recall, F1 score, and the confusion matrix heat map. Accuracy represents the percentage of correctly categorized examples out of the total number. Precision measures a model’s ability to distinguish between positive and negative samples by calculating the ratio of correctly predicted positive samples. Recall quantifies the percentage of positive samples correctly predicted in a given sample set, with higher recall values indicating models that excel at identifying positive samples. The F1 score provides a balanced metric that considers both precision and recall. The confusion matrix heat map visualizes the prediction result of the classification model and facilitates analysis of its strengths and weaknesses. Detailed formulas for these indicators are provided in Equations (3)–(6):

(3) $$Recall=\frac{TP}{TP+FN},$$

(4) $$Precision=\frac{TP}{TP+FP},$$

(5) $$Accuracy=\frac{TP+TN}{TP+TN+FP+FN},$$

(6) $$F1_{score}=2\times \frac{Precision\times Recall}{Precision+Recall},$$

where *TP* is the number of positive samples that are correctly predicted, *FP* is the number of negative samples that are incorrectly predicted, *TN* is the number of negative samples that are correctly predicted, and *FN* is the number of positive samples that are incorrectly predicted.

### 2.6. Experimental Environment

The training process for the proposed model was run on the Linux operating system and PyTorch2.0.0 (GPU version) framework. The software environment was CUDA11.8 and Python 3.8. The CPU used for the training data set was a 12 vCPU Intel(R) Xeon(R) Silver 4214R CPU @ 2.40 GHz, and the GPU was an RTX 3080 Ti (12 GB). The batch size was set to 16, and the number of iterations was set to 50.

## 3. Results

### 3.1. Application of Five Lightweight Models for Wheat Disease Identification

**Figure 5:**
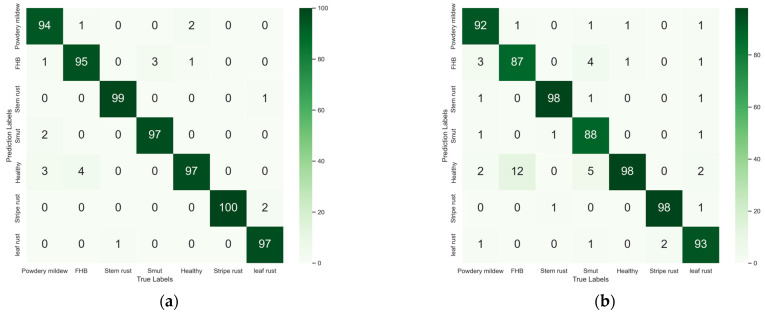

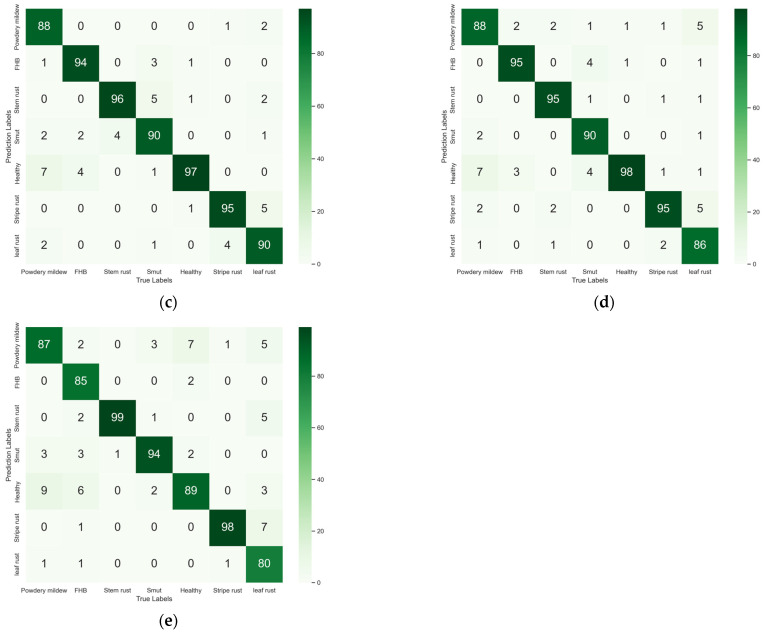
Confusion matrices of five classification models on the test set: (**a**) Eficientnetv2; (**b**) MnasNet; (**c**) MobileNetV3; (**d**) GhostNet; and (**e**) ShuffleNetV2.

The SGD and Adam optimizers were used to train five lightweight models with different initial learning rates, and the accuracy and loss values of the models were recorded. The three optimal weight data sets were used to calculate the average accuracy and standard error. The results of the five models regarding the recognition of six different wheat diseases and healthy wheat were compared. The training weight parameter sizes are provided in Table 2. The training results for the five lightweight models are listed in Table 3, and the accuracy and loss values are shown in Figure 5.

As shown in Table 3, there were significant differences in average test accuracy of the same model under different initial learning rates and different training strategies (*p* < 0.05). Therefore, an appropriate initial learning rate needs to be chosen, according to the specific task. The five lightweight models all showed good recognition effects, and the accuracy rate reached 90% on the test set. Among them, the model with the highest accuracy on the test set was efficientnetv2, which achieved 97%, followed by MnasNet and GhostNet, which reached 93.43% and 93.29%, respectively. The results showed that the use of a lightweight convolutional neural network for wheat disease identification in the actual field environment is feasible. MnasNet had smaller model parameters and, thus, trained faster: in particular, its parameter size was 19.11 MB, making it approximately four times smaller than the EfficientNetV2 model. Therefore, MnasNet was used for follow-up improvement and optimization.

Figure 4 shows the confusion matrix for the five models. The darker the color of diagonal elements, the better the recognition performance of the model. EfficientNetV2 had the best identification performance in all seven categories, while the MnasNet model had similar performance regarding the identification of different diseases.

### 3.2. Influence of SimAM Attention Mechanism on MnasNet Model

λ is an important parameter in the SimAM module that affects the identification accuracy of the model. In this study, λ values were set in the range of 10^−3^ to 10^−7^, and a comparative analysis was performed. The average and maximum accuracies of the results from different λ values on the test set are shown in Table 4. Each λ value was repeated three times, and the mean value and standard error were obtained. The optimal SimAM module was selected for comparison with other attention mechanisms (CA, ECA, SE, CBAM) on the wheat disease data set.

As shown in Table 4, the model’s recognition effect was better when λ = 10^−5^ or 10^−6^. This demonstrates that the model paid more attention to the detailed characteristics of samples when the λ value was small, which were taken as an important basis to judge the disease. The model could more accurately distinguish different types of diseases through analyzing the local characteristics of the samples. When the λ value was 10^−7^, the accuracy of the model decreased, as a smaller λ value led to a narrower search range, such that only the local features were focused on while the global information was ignored.

**Figure 6:**
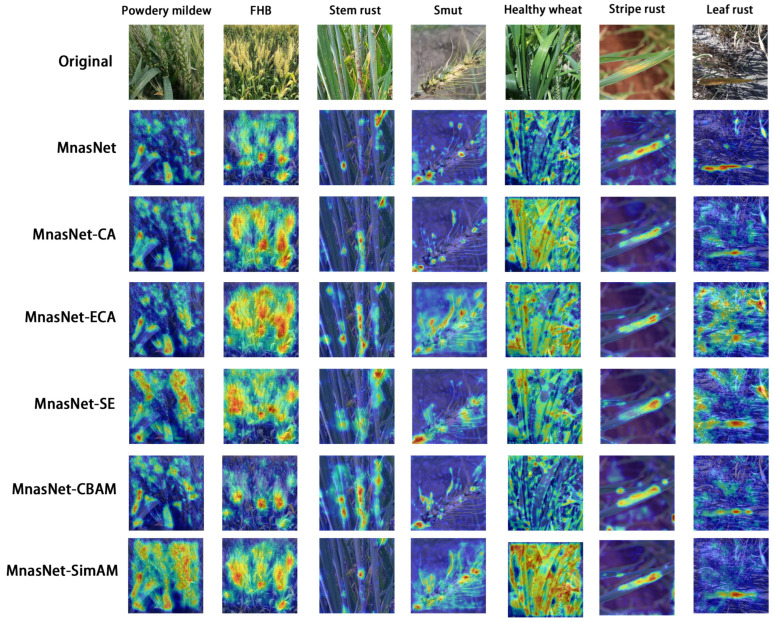
Class activation diagram for MnasNet with different attention mechanisms.

From Table 5, it can be seen that the accuracy of the model increased by 1.14%, 1.42%, 0.57%, 0.42%, and 1.71% after the addition of CA, ECA, SE, CBAM, and SimAM attention modules, respectively. MnasNet-SimAM had the best accuracy, at 95.14%. In this study, the Grad-Cam class activation diagram (Figure 6) was used to visualize the degree of attention paid to lesion features by the last three inverted residual networks of the model after the addition of attention mechanisms. It can be seen that MnasNet-SimAM was much more focused on the lesion site than the original model. In addition, the influence of some complex and irrelevant background elements on the classification task was reduced. Particularly in the classification of wheat smut, MnasNet not only expanded the focus on disease spots, but also on small disease spots that were overlooked by the original model. Table 6 provides the classification results of MnasNet-SimAM for common wheat diseases. With F1 scores used as the final evaluation index, the recognition performance of the model reached more than 90% for each category, and the F1 score for wheat stripe rust reached 98%.

### 3.3. Effect of Dual Transfer Learning Using Agricultural Disease Pre-Training Weights

**Figure 7:**
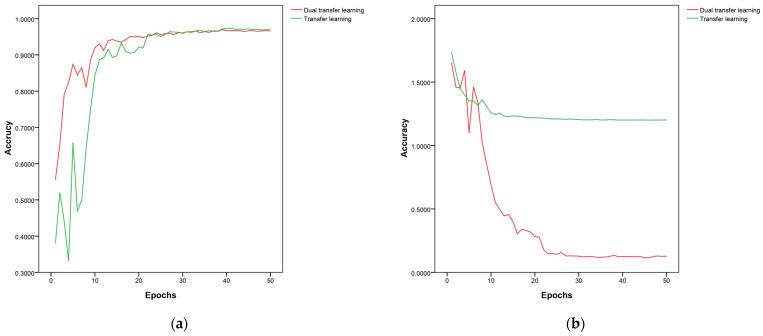
(**a**,**b**) Comparison of accuracy and loss of dual transfer learning and single transfer learning on validation set.

**Figure 8:**
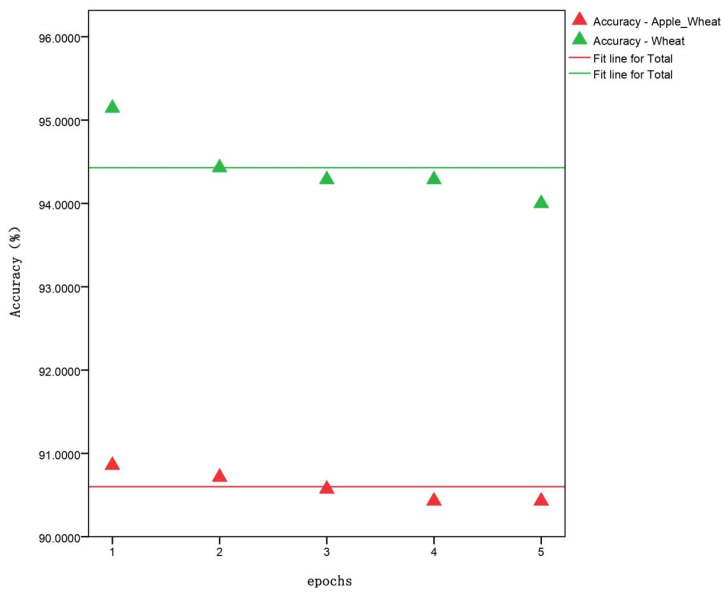
Scatter plots of the highest quintic precision with dual transfer learning and single transfer learning.

Next, the influence of dual transfer learning on wheat disease recognition based on agricultural pre-training weights was explored. The weights with the highest accuracy obtained through MnasNet-SimAM dual transfer learning were saved, and the changes in accuracy and loss values and the comparison with single transfer learning were recorded, as shown in Figure 7. The results show that, when using dual transfer learning, a higher initial accuracy and lower losses were achieved, compared to single transfer, with the validation set having only one-tenth of the losses observed with single transfer learning. When the model was trained to converge at 25 epochs, using dual transfer learning reduced the training time by 2.566 min compared to single transfer, which accounted for 16.81% of the convergence process. Figure 8 exhibits the accuracy of the top five training weights in the test set for dual transfer learning versus single transfer learning. Dual transfer learning for training improved the speed of convergence on the validation set, but decreased it on the test set by about 4%. When trained on the apple leaf disease data set, the model might overfit the specific features of the data set, leading to a decrease in its generalization ability on the wheat disease data set.

### 3.4. Testing Model Robustness on a Public Data Set

**Figure 9:**
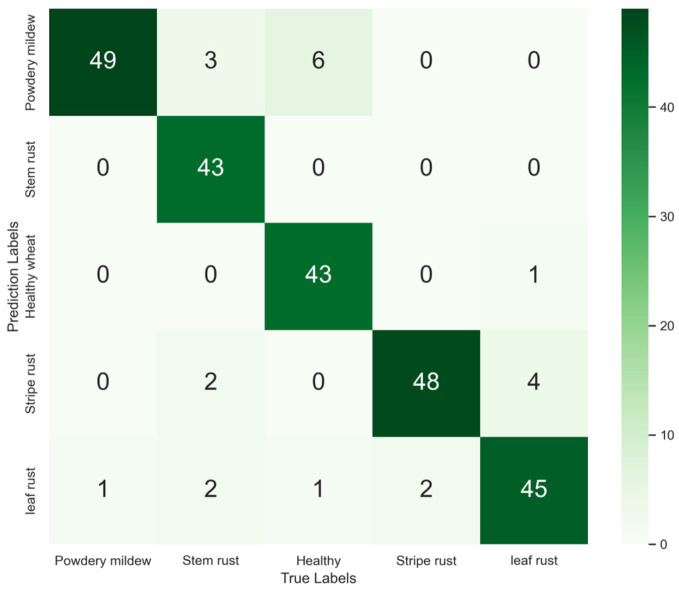
Confusion matrix of MnasNet-SimAM on the WFD test set.

The Wheat Fungi Diseases (WFD) data set (available at http://wfd.sysbio.ru/index.html accessed on 6 August 2024) includes wheat stripe rust, leaf rust, stem rust, powdery mildew, and healthy leaf images. For this experiment, 50 images for each disease category were randomly selected from these data, and a total of 250 images were used to construct the WFD test set. The WFD test set was then used to evaluate the MnasNet-SimAM model (see Figure 9 and Table 7).

The obtained results indicated that MnasNet-SimAM had better fitting ability and robustness, with accuracy of 91.20% on the public data set. The F1 scores for the five categories were as follows: 90.74%, 92.47%, 92.47%, 92.31%, and 89.11%.

## 4. Discussion

Many studies have shown that the use of attention mechanisms can significantly improve the performance of models. As this study was based on transfer learning to build a wheat disease recognition network, the addition of an attention mechanism should not change the model network structure. Therefore, the SimAM module was added to the last three layers of the inverted residual network of MnasNet. The resulting model could capture more global context information and had improved understanding of the input image. The non-linear relationships between pixels could be better captured by this model. The complex features of the image could be better extracted, and useful information could be obtained while suppressing useless information [38,39,40]. The size of the original model only increased by 0.01 MB after adding the SimAM module, meaning that it remained efficient for training agricultural disease image classification models. Li [41] introduced a convolutional neural network model called Sim-ConvNeXt for maize disease classification. The SimAM attention module was integrated into this model, and the accuracy was improved by 1.5% based on the original model, consistent with the results of this study.

λ is an important parameter used by the SimAM module to calculate the importance of neurons. It is a regularization term that is used to add a small constant to the denominator when calculating the variance, thus ensuring numerical stability and avoiding division by zero. Yang [18] explored the influence of the λ value on the SimAM module performance, and the highest accuracy was achieved with a value of 10^−5^, while the performance declined with a value of 10^−6^. However, in this study, the average accuracy on the test set was similar when the λ value was equal to 10^−5^ or 10^−6^. Different from the results obtained by Yang, the maximum accuracy of the module was 95.14% when λ was equal to 10^−6^. The strength of the attention mechanism and the performance of the model may be differently affected by the λ value due to differences in the nature of the task. In this study, the attention mechanism almost failed when λ was close to zero. The model might ignore most of the information in the input data, such that its focus on important features was lost, degrading the model’s performance. When λ was too large, the SimAM module focused too much on some local features and ignored others, causing the model to be overly sensitive and/or overfit to noise and irrelevant information. The optimal value of the parameter λ can be expected to vary, according to the global or local attention required in the actual task. Therefore, determination of the parameter λ could require multiple tests and different hyperparameter adjustments. In the training of models for agricultural disease classification tasks, the size of λ needs to be reduced such that the model can pay attention to the small disease spots while ignoring the influence of the complex background.

Dual transfer learning is an advanced transfer learning technology that allows for better adaptation to the target task through the use of knowledge from multiple source tasks simultaneously. Zhao [25] investigated the impact of dual transfer learning on ResNet50. The ImageNet data set was used for single transfer learning, while the AI Challenger 2018 data set was used for dual transfer learning. Dual transfer learning improved the model’s training efficiency and accuracy. As mentioned in the study of Mukhlif [37], most of the previous transfer learning studies suffer from overfitting; hence, a 50% dropout layer was added to their experiments to minimize this problem. In this study, we also increased the dropout layers by 20%, but the accuracy on the test set still decreased by about 4%. This might be due to domain differences, feature mismatches, or overfitting, as reported in previous studies. Although this sped up the training convergence time, it is not cost-effective to sacrifice accuracy with high computing power and small samples. Therefore, researchers need to continue to explore ways in which training speed can be improved while maintaining accuracy in the future.

When tested on the WFD data set, the F1 score for wheat leaf rust was below 90%. This was because 4 out of 50 images of leaf rust were misclassified as stripe rust. In the study of Jiang [42], the seven models tested also misjudged stripe rust and leaf rust in the wheat disease recognition task. The most serious misjudgment (at 8%) was observed with DenseNet-121. As a certain similarity between wheat stripe rust and leaf rust in disease spots could lead to model classification errors, we should consider how to distinguish similar diseases in future work.

## 5. Conclusions

This study explored the possibility of using lightweight convolutional neural networks to recognize common wheat diseases based on transfer learning. A model named MnasNet-SimAM was constructed based on the SimAM attention mechanism. The proposed model achieved a highest accuracy of 95.14% and an average accuracy of 94.62% on the test set. Moreover, the parameter size of the improved model was only increased by 0.01 MB, while the accuracy was improved significantly (by 1.7%) over that of the original model. Furthermore, the use of pre-training weights for dual transfer learning sped up model convergence and reduced convergence time by 16.81%. On the Wheat Fungi Diseases data set, MnasNet-SimAM achieved an accuracy of 91.20%, indicating that MnasNet-SimAM is robust and can be used for wheat disease identification on mobile devices.

## Figures and Tables

**Table 1:** Sample distribution in wheat disease data sets.

Type	Training Set	Validation Set	Test Set
Wheat leaf rust	700	200	100
Wheat stem rust	700	200	100
Wheat stripe rust	700	200	100
Wheat powdery mildew	700	200	100
FHB in wheat	700	200	100
Wheat smut	700	200	100
Healthy wheat	700	200	100
Total	4900	1400	700

**Table 2:** Training weight parameter size of the five lightweight models.

Model	Transfer Learning	Parameter Size (MB)
EfficientNetv2	Yes	77.01
MnasNet	Yes	19.11
GhostNet	Yes	14.92
MobileNetV3	Yes	16.06
ShuffleNetV2	Yes	20.44

**Table 3:** Results for five lightweight models under different learning rates (*p* < 0.05).

Model	Initial Learning Rate	Scheduling Strategy	Average Train Accuracy (%)	Average Verification Accuracy (%)	Average Test Accuracy (%)	Maximum Test Accuracy (%)
MnasNet	0.0001	Adam	99.87 ± 0.04	95.81 ± 0.11	92.10 ± 0.19 bc	92.29
	0.0001	Adam + StepLR	99.96 ± 0.00	97.59 ± 0.07	93.00 ± 0.14 a	93.14
	0.0001	Adam + Wramup + COS	99.98 ± 0.00	97.09 ± 0.04	91.62 ± 0.34 c	92.29
	0.001	SGD	99.82 ± 0.01	97.47 ± 0.02	93.24 ± 0.13 a	93.43
	0.001	SGD + StepLR	99.81 ± 0.02	97.59 ± 0.07	92.62 ± 0.13 b	92.86
	0.001	SGD + Wramup + COS	99.99 ± 0.01	97.44 ± 0.00	93.00 ± 0.00 a	93.00
EfficientNetNetv2	0.0001	Adam	99.83 ± 0.05	97.32 ± 0.06	94.00 ± 0.22 c	94.43
	0.0001	Adam + StepLR	99.92 ± 0.00	97.47 ± 0.06	94.43 ± 0.00 bc	94.43
	0.0001	Adam + Wramup + COS	99.96 ± 0.00	98.06 ± 0.02	94.95 ± 0.10 b	95.14
	0.01	SGD	99.89 ± 0.01	97.32 ± 0.05	93.67 ± 0.31 c	94.29
	0.01	SGD + StepLR	99.98 ± 0.00	97.82 ± 0.06	96.62 ± 0.19 a	97.00
	0.01	SGD + Wramup + COS	99.98 ± 0.00	97.04 ± 0.02	95.10 ± 0.05 b	95.14
MobileNetV3	0.0001	Adam	99.84 ± 0.03	96.92 ± 0.06	90.05 ± 0.05 bc	90.14
	0.0001	Adam + StepLR	99.91 ± 0.02	97.06 ± 0.02	89.52 ± 0.37 c	90.14
	0.0001	Adam + Wramup + COS	99.95 ± 0.01	96.33 ± 0.09	86.62 ± 0.34 d	87.29
	0.01	SGD	99.81 ± 0.01	97.04 ± 0.02	90.67 ± 0.42 b	91.43
	0.01	SGD + StepLR	99.98 ± 0.01	97.28 ± 0.09	92.52 ± 0.21 a	92.86
	0.01	SGD + Wramup + COS	99.97 ± 0.01	97.35 ± 0.02	89.86 ± 0.14 bc	90.14
GhostNetV1	0.0001	Adam	99.27 ± 0.07	94.74 ± 0.07	89.67 ± 0.10 bc	89.86
	0.0001	Adam + StepLR	99.88 ± 0.05	95.90 ± 0.04	88.48 ± 0.19 d	88.86
	0.0001	Adam + Wramup + COS	99.95 ± 0.01	96.21 ± 0.04	88.76 ± 0.33 cd	89.29
	0.01	SGD	99.82 ± 0.01	96.64 ± 0.15	92.19 ± 0.55 a	93.29
	0.01	SGD + StepLR	99.96 ± 0.02	96.64 ± 0.04	89.71 ± 0.25 b	90.14
	0.01	SGD + Wramup + COS	99.95 ± 0.01	96.43 ± 0.04	92.24 ± 0.10 a	92.43
ShuffleNetV2	0.0001	Adam	99.87 ± 0.02	96.57 ± 0.10	82.48 ± 0.29 c	83.00
	0.0001	Adam + StepLR	99.95 ± 0.01	96.16 ± 0.04	88.10 ± 0.19 b	88.29
	0.0001	Adam + Wramup + COS	99.98 ± 0.01	93.25 ± 0.08	68.95 ± 0.25 e	69.43
	0.001	SGD	99.86 ± 0.03	96.16 ± 0.04	83.14 ± 0.46 c	84.00
	0.001	SGD + StepLR	99.84 ± 0.01	96.85 ± 0.02	77.14 ± 1.25 d	79.43
	0.001	SGD + Wramup + COS	99.98 ± 0.00	97.11 ± 0.02	90.29 ± 0.00 a	90.29

Note: Different lowercase letters indicated the significant difference at the 0.05 level.

**Table 4:** Influence of different λ values on accuracy (*p* < 0.05).

λ Value	Average Test Accuracy (%)	Maximum Test Accuracy (%)
10^−3^	89.62 ± 0.05	89.71
10^−4^	93.10 ± 0.05	93.14
10^−5^	94.71 ± 0.14	95.00
10^−6^	94.75 ± 0.20	95.14
10^−7^	91.90 ± 0.13	92.14

**Table 5:** Comparison of different attention mechanisms with the original model on the test set (*p* < 0.05).

Network	Average Test Accuracy (%)	Maximum Test Accuracy (%)	Parameter Size (MB)
MnasNet	93.24 ± 0.13	93.43	19.11
MnasMet-CA	94.38 ± 0.13	94.57	19.24
MnasMet-ECA	94.62 ± 0.13	94.85	19.12
MnasMet-SE	93.81 ± 0.10	94.00	19.24
MnasMet-CBAM	93.71 ± 0.08	93.85	19.29
MnasMet-SimAM	94.62 ± 0.27	95.14	19.12

**Table 6:** Classification results of the final model.

Diseases of Wheat	Accuracy (%)	Precision (%)	Recall (%)	F1 Score (%)
Powdery mildew	95.14%	87.50	98.00	92.45
FHB		96.00	96.00	96.00
Stem rust		98.92	92.00	95.34
Smut		94.00	94.00	94.00
Healthy wheat		98.96	94.06	96.45
Stripe rust		98.00	98.00	98.00
Leaf rust		93.94	93.00	93.47

**Table 7:** Classification results of MnasNet-SimAM on the WFD data set.

Diseases of Wheat	Accuracy (%)	Precision (%)	Recall (%)	F1 Score (%)
Powdery mildew	91.20%	84.48	98.00	90.74
Stem rust		100.00	86.00	92.47
Healthy wheat		97.73	87.76	92.47
Stripe rust		88.89	96.00	92.31
Leaf rust		88.24	90.00	89.11

## Data Availability

Data are contained within the article.
